# Dengue vectors in Africa: A review

**DOI:** 10.1016/j.heliyon.2022.e09459

**Published:** 2022-05-17

**Authors:** Diawo Diallo, Babacar Diouf, Alioune Gaye, El hadji NDiaye, Ndeye Marie Sene, Ibrahima Dia, Mawlouth Diallo

**Affiliations:** Pôle de Zoologie Médicale, Institut Pasteur de Dakar, 36 Avenue Pasteur, BP 220, Dakar, Senegal

**Keywords:** Dengue, Mosquito vectors, Sylvatic, Urban, Africa

## Abstract

Dengue fever is a mosquito-borne-disease of growing public health importance in Africa. The continuous increase of number and frequency of outbreaks of dengue fever, especially in urban area in Africa underline the need to review the current data available on vectors involved in dengue virus transmission in Africa. Here, we summarized the available data on vectors involved in the transmission of dengue virus in the sylvatic and urban environments, vertical transmission, vector competence studies, and vector control strategies used in Africa. The virus was isolated mainly from *Aedes furcifer*, *Ae. luteocephalus*, and *Ae. taylori* in the sylvatic environment and from *Ae. aegypti* and *Ae. albopictus* in the urban areas. Prospective and urgently needed studies on vectors biology, behavior, and alternative control strategies are suggested.

## Graphical abstract

**Figure:**
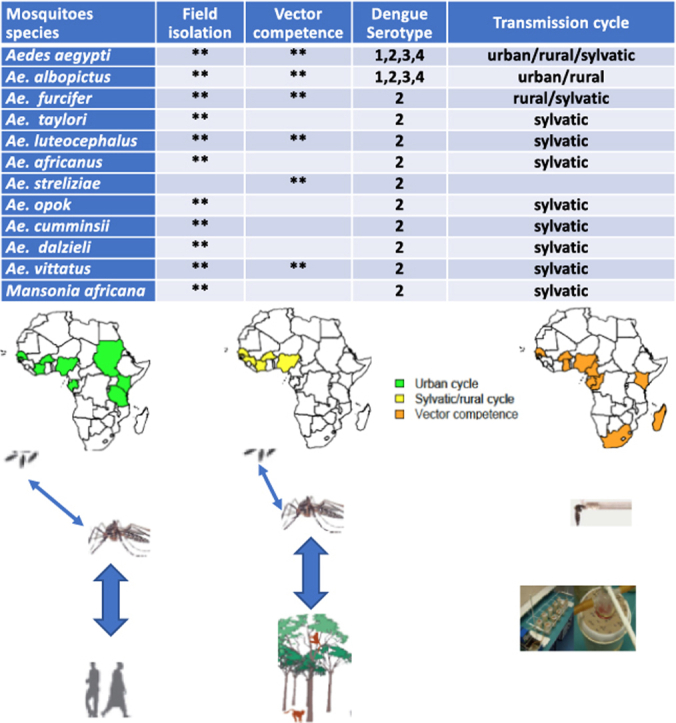

## 1 Introduction

**Figure 1:**
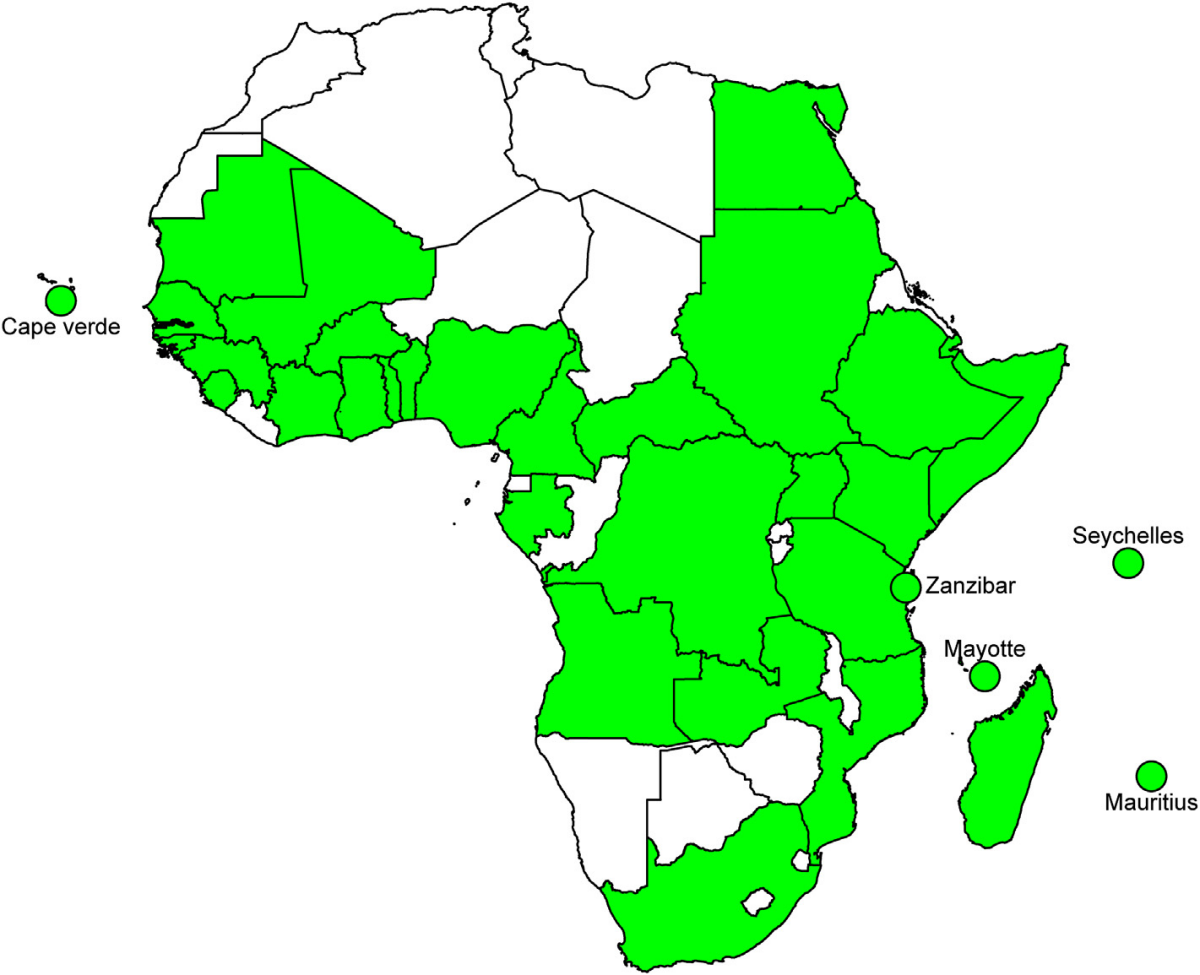
Map showing African countries where dengue circulation has been detected in local populations or travelers.

Dengue virus (DENV), the causative agent of Dengue fever, transmitted by several *Aedes* spp., is one of the most important arboviral diseases in the world. Indeed, this disease caused by four genetically related but antigenically distinct viruses (DENVs 1–4; genus *Flavivirus*, family *Flaviviridae*), is endemic in more than 128 countries with around 390 million people infected each year [1, 2]. With 16% of these infections, Africa is one of the most affected regions [3]. Evidence of dengue circulation has been detected in local populations or travelers returning from more than 30 African countries (Figure 1).

In Africa, DENV-2 was the most frequently reported serotype before 2000s and was responsible of several epidemics in East Africa (Somalia, Djibouti, Kenya, and Tanzania) and sylvatic emergence with sporadic human cases in West Africa (Senegal, Burkina and Côte d’Ivoire) [4, 5, 6, 7, 8]. In West Africa, DENV-2 epidemics were only detected in Nigeria and Burkina Faso before 2000 [9,10]. The DENV-3 emerged only in Mozambique in 1985 and Somalia in 1993 [8,11], while DENV-1 outbreaks were observed in Sudan in 1984, Comoros in 1993, Nigeria in 1960s and Senegal in 1979 [6,9,12]. In recent decades, the number and frequency of dengue epidemics have increased dramatically in Africa [13, 14, 15]. DENV-2 and 3 are the main serotypes involved in the epidemics on the African continent, although the circulation of the other two serotypes have been documented [16]. In East Africa, DENV-2 has remained very active in countries that were affected during the last century, but has emerged also in several other countries, including Ethiopia in 2013, Tanzania in 2014 and Mozambique in 2014–2015 [17,18,19,20]. Sylvatic amplification and urban epidemics of DENV-2 have occurred in several West African countries (Mali in 2008, Senegal in 2015–2018, Mauritania in 2014–2020, and Burkina Faso in 2013, 2016 and 2017), as well as in Central African countries (Gabon in 2007 and Angola in 2013 and 2018) [13,21,22,23,24]. Outbreaks of DENV-3 have been reported in Tanzania, Zanzibar, Comoros, Benin, Cape Verde, Gabon and Senegal in 2009–2018 [25,26,27,28]. Epidemics of DENV-1 were detected in Angola, Kenya, Senegal and Somalia in 2011–2018 [17,29,30,31].

Because dengue is becoming a major threat to public health in Africa, it is essential to better understand some poorly characterized aspects of the mosquito vectors involved in the epidemiology and transmission of this disease. Since there is no specific treatment or available vaccine for dengue, a better understanding of these factors will be a key driver for designing effective and sustainable vector control methods and strategies.

The goal of this review is to compile the available data on the dengue virus vectors in Africa based mainly on virus isolation from field collected mosquitoes and vector competence studies.

## 2 Mosquito species associated to dengue virus in the field

Several mosquito species have been associated with DENV in field collected mosquitoes from both urban and sylvatic environments (Table 1).

**Table 1:** Mosquito species found naturally infected with dengue virus in Africa.

Environment	Countries	Species	Years	Serotypes	References
Urban	Senegal	*Ae. (Stegomyia) aegypti*	2009	3	[27]
	Burkina Faso		1982, 1986	2	[59]
	Nigeria		1969	2	[41, 42, 57]
	Côte d’Ivoire		2017	3	[46]
	Tanzania		2014	2	[19]
	Kenya		2014	2	[47]
	Sudan		2016–17	??	[48]
	Cabo Verde		2009, 2014-15	2, 3, 4	[45]
	Seychelles	*Ae. (Stegomyia) albopictus*	1976–77	2	[51]
	Gabon		2007, 2010	2	[23, 50]
Rural/Sylvatic	Senegal	*Ae. (Stegomyia) aegypti*, *Ae. (Diceromyia) furcifer*, *Ae. (Diceromyia) taylori*, *Ae. (Stegomyia) luteocephalus*, *Ae. (Aedimorphus) dalzieli*, *Ae. (Aedimorphus) vittatus*, *Ma. (Mansonoides) africana*	1974, 1981–82, 1989, 1999–2000	2	[4,53,84]
	Burkina Faso	*Ae. (Stegomyia) luteocephalus*, *Ae. (Stegomyia) africanus*, *Ae. (Aedimorphus) cumminsii*	1980, 1983, 1986	2	[44, 59]
	Côte d’Ivoire	*Ae. (Stegomyia) aegypti*, *Ae. (Diceromyia) furcifer*, *Ae. (Diceromyia) taylori*, *Ae. (Stegomyia) luteocephalus*, *Ae. (Stegomyia) africanus*, *Ae. (Stegomyia) opok*	1980, 1985-87	2	[4, 59]
	Nigeria	*Ae. (Stegomyia) luteocephalus*, *Ae. (Stegomyia) africanus* *Ma. (Mansonioides) africana*	1969, 1977	1, 2, 3	[60, 61]
	Guinea Conakry	*Ae. (Stegomyia) luteocephalus*, *Ae. (Stegomyia) africanus*	1981	2	[4]

### 2.1 Vectors in the urban environment

In Africa, DENV is transmitted in the urban environment, between humans and the mosquito species *Ae. aegypti* and *Ae. albopictus* during epidemics. In urban environments, *Aedes* vectors were found breeding indoors and outdoors in human associated water storage containers (clay jars, drums, jerrycans, cement tanks, etc), discarded containers, used tires, flower pots, miscellaneous, etc. [32, 33, 34, 35]. These *Aedes* are mainly anthropophilic, but were also found to have fed on other animals [36, 37, 38]. *Aedes aegypti* is considered as mainly endophilic, endophagic, and daytime feeder, but it was collected feeding and resting outdoors within used tires, bricks and scrap metals indicating that it can also transmit viruses outdoors [39]. Alternatively, *Ae. albopictus* is considered as being a more opportunistic and outdoor feeder [37, 38]. Climatically suitable areas for *Ae. aegypti* related to dengue incidence was predicted to increase in the future [40]. These authors found that temperature and precipitation (by providing breeding sites and stimulates egg hatching) are important climatic factors that will influence *Ae. aegypti* development and distribution.

**Figure 2:**
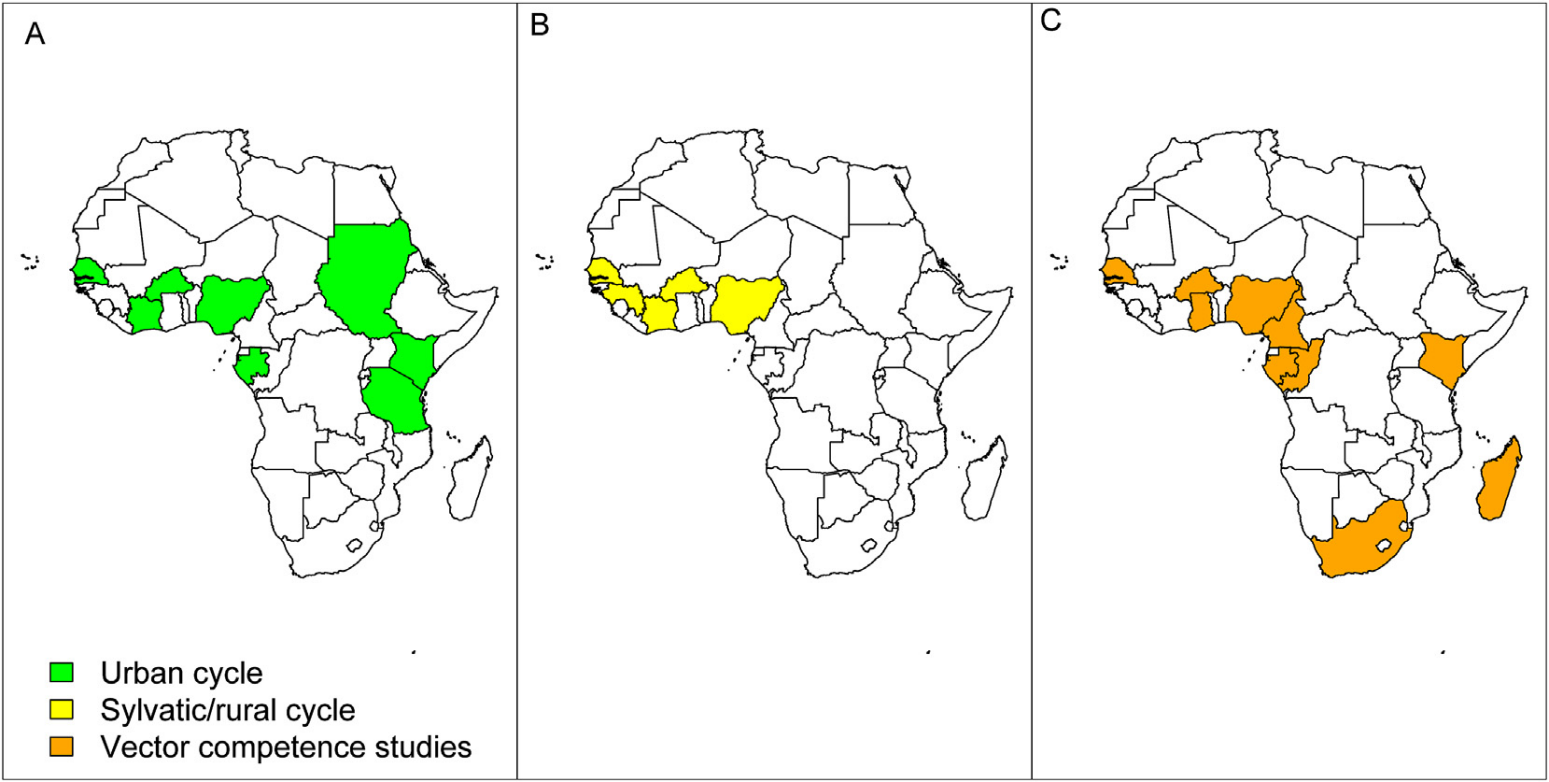
Map showing African countries where dengue virus was detected from mosquitoes in the urban (A) and sylvatic/Rural cycle (B), and vector competence studies (C) were done.

The DENV was associated with *Ae. aegypti* in an urban cycle, in Senegal, Nigeria, Burkina Faso, Cabo Verde, Tanzania, Kenya and Sudan during outbreak investigations and/or routine entomological studies (Figure 2). Despite its presence in several African countries, Gabon is actually the only country where DENV is associated with *Ae. albopictus* in continental Africa.

In Nigeria, *Ae. aegypti* and *Ae. albopictus* have been collected during entomological studies but only *Ae. aegypti* was found infected with all DENV serotypes in the field [41, 42]. A pool of *Ae. aegypti* collected in August 1969 in Ibadan was found positive for DENV-2. Viral RNAs of the four DENV serotypes were detected in *Ae. aegypti* collected in different localities of Nigeria in 2001–2002. Three pools of *Ae. aegypti* were found infected in 2 neighborhoods of Dakar (2 pools in Plateau and 1 pool in Parcelles Assainies), during the first urban epidemic of DENV-3 in Senegal [27]. The *Stegomyia* indexes in Dakar were above the epidemic risk threshold (between 6.6-195.2 for the Breteau index and 15–63.2 for the container index). *Aedes aegypti* was also the only potential vector present in high abundance in all the following urban epidemics and sporadic cases of dengue in 2015 (DENV-2; Mbour), 2017 (DENV-3; Louga) and 2018 (DENV-1 and 2 in Fatick; DENV-1 and 3 in Touba; DENV-1 in St Louis) that occurred in Senegal [28, 31, 43]. In Burkina Faso, DENV was isolated from *Ae. aegypti* in 1982 (1 pool) and 1986 (1 pool) during DENV-2 epidemics in the urban area of Bobo-Dioulasso [44]. During the first dengue epidemic that occurred in Cabo Verde in 2009, DENV-3 was isolated from a pool of *Ae. aegypti* collected at Praia, the capital city of the country. Two other serotypes (3 strains of DENV-2 and 5 strains of DENV-4) were later detected from the same country by RT-PCR from *Ae. aegypti* mosquitoes collected in 2014 and 2015, in Palmarejo (7 strains) and Fonton (1 strain), 2 neighborhoods of Praia city [45]. DENV-3 was detected by RT-PCR from 3 *Ae. aegypti* pools (2 from host-seeking females and 1 from emerging adults collected as larvae) collected at Abidjan, the economic capital city of Côte d’Ivoire, during the 2017 outbreak [46]. The Breteau (213 and 297), House (69 and 82%) and containers (35 and 41%) indexes were high in the 2 localities investigated during this outbreak.

The first association of DENV with *Ae. aegypti* in Tanzania was detected by an entomological investigation during the 2014 dengue outbreak [19]. DENV-2 was detected in 8.2% of 330 *Aedes* mosquito pools tested by RT-PCR. Only 2 out of the 27 positives *Ae. aegypti* pools were collected as adults. The *Aedes* indices (Breteau index: 20.8–30.6 and container index: 65.2–80.2%) were very high, suggesting high risks of infection in all the districts investigated within Dar es Salaam. DENV-2 was isolated from a pool of 2 males *Ae. aegypti* collected during an outbreak in Mombasa in 2013–2014, suggesting that this species was the vector of this urban epidemic in eastern Kenya [47]. The *Aedes* indices were all high in the study area (Breteau index: 123.1–358.6 and container index: 23.3–44.1%). During a routine entomological investigation in Kassala States in eastern Sudan, DENV RNA was detected in one out of 329 *Ae. aegypti* adults tested by RT-PCR. This species was the only vector identified during this study. The house and Breteau index were 32.8 % and 35.96, respectively [48]. The serotype was not identified during this outbreak but all dengue serotypes have been previously detected in Sudan. DENV-1 and DENV-2 were the most commonly found in this country [48, 49].

While both *Ae. aegypti* and *Ae. albopictus* were collected during a DENV-2 outbreak in Gabon in 2007 [23], the virus was only detected in *Ae. albopictus* (3 pools) in the suburbs of the capital city, Libreville, suggesting *Ae. albopictus* was the primary vector during this epidemic. Importantly, this was the first detection of DENV in *Ae. albopictus* in continental Africa and *Ae. albopictus* was more abundant than *Ae. aegypti* in this suburban environment. *Aedes albopictus* was also the vector of a second DENV-2 outbreak in Franceville, in Gabon in 2010 when the virus was detected in 18 out of 46 pools of this species tested [50]. One female was found coinfected with dengue and chikungunya viruses during this outbreak. Prior to the Gabon outbreaks, DENV-2 was isolated from 8 pools of *Ae. albopictus* collected during a dengue outbreak in the Seychelles in 1976–1977 [51]. Although multiple other dengue fever outbreaks have occurred in several other African countries, data on which mosquito species were involved is lacking because no entomological investigation was carried out during these outbreaks [4, 13, 52].

### 2.2 Vectors in the sylvatic and rural environments

Detection of DENV from several mosquito species (*Ae. furcifer*, *Ae. taylori*, *Ae. luteocephalus*, *Ae. vittatus*, *Ae. africanus*, *Ae. opok*, *Ae. cumminsii*, *Ae. dalzieli*, and *Ae. aegypti*) in sylvatic and rural areas follows a seasonal pattern. Dengue virus has mainly been detected between August and November in forest galleries and villages [53]. Some of these species (*Ae. furcifer*, *Ae. taylori*, *Ae. luteocephalus*, *Ae. africanus* and *Ae. opok*) are known to be crepuscular feeders [54]. Moreover, depending on the species considered, several of them (*Ae. furcifer*, *Ae. taylori*, *Ae. luteocephalus*, *Ae. africanus*, and *Ae. opok*) seem to feed almost exclusively on human and or non-human primates. Although they are found readily feeding on men, *Ae. vittatus, Ae. cumminsii*, *Ae. dalzieli*, sylvatic *Ae. aegypti*, and *Ma. africana* are considered as mainly zoophilic [55]. Some species like *Ae. luteocephalus* and *Ae. taylori* feed mainly in the forest-canopy, while others could be found feeding in almost all landcover classes found in the sylvatic environment, including villages (outdoors and indoors) and agricultural areas where feeding on humans is possible [56]. *Aedes furcifer* females infected with DENV were collected within villages investigated in southeastern Senegal from 2009 to 2020, suggesting that they are involved in DENV-2 transmission to humans in this area [53]. The main breeding places are tree holes and fruit husks for most sylvatic vectors (*Ae. furcifer*, *Ae. taylori*, *Ae. luteocephalus*, *Ae. africanus*, and *Ae. opok*), rock pools and puddles for *Ae. vittatus* [32], ground pools and puddles for *Ae. dalzieli* and *Ae. cumminsii*, and grassy ponds for *Ma. africana*.

The sylvatic cycle of DENV has been described in five West African countries and mainly for DENV-2 (Table 1 and Figure 2) [53]. The first isolations of DENV-2 from naturally infected mosquitoes in Africa date to 1969 when one strain was isolated most likely from *Ae. (Stegomyia) luteocephalus* in Jos in Nigeria [57]. Later, DENV-2 was isolated from mosquitoes collected in wooded areas in Senegal [53, 58], Côte d’Ivoire [59], Guinea [4] and Burkina Faso [44]. The virus was isolated mainly from *Ae. luteocephalus*, *Ae. furcifer* and *Ae. taylori* in Senegal and Côte d’Ivoire, and *Ae. luteocephalus* in Burkina Faso. Several other species were sporadically found associated with DENV in the sylvatic environment in Senegal (*Ae. aegypti, Ae. vittatus, Ae. dalzieli, Ma. africana*), Burkina Faso (*Ae. africanus*, *Ae. cumminsii)* and Côte d’Ivoire (*Ae. africanus*, *Ae. opok*, *Ae. cumminsii*). DENV-2 was isolated from 4 pools of mosquitoes (3 *Ae. africanus* and 1 *Ae. luteocephalus*) collected in November 1981 in Guinea [4].

DENV-1 was isolated from two pools of *Ae. africanus* collected in the Mamu River Forest reserve in Eastern Nigeria in 1997 [60]. DENV-3 viral RNA was also detected in 30 pools of *Ma. africana* collected in the rural locality of Ikarama, state of Bayelsa in Nigeria in 2015 [61]. The real importance of *Ae. africanus* and *Ma. africana* in the transmission of dengue in the rural human dwelling area need further studies.

## 3 Vertical transmission studies

In Africa, DENV is likely maintained in nature during unfavorable periods, by vertical transmission as suggested by detection of the virus from male mosquitoes, adults collected as immatures during field studies and progeny of infected females. Indeed, DENV was detected from male mosquitoes belonging to several species including *Ae. aegypti* in Nigeria [41] and Kenya [47], *Ae. furcifer-taylori* in Côte d’Ivoire in 1980 [59], *Ae. taylori* [62] and *Ae. furcifer* in a forest gallery in Kedougou [53] and unidentified *Aedes* species in Nigeria [41]. Most of the DENV-2 positive pools of *Ae. aegypti* (25 out of 27), detected by RT-PCR during the 2014 dengue outbreak in Dar es Salaam, the capital city of Tanzania were collected as immatures [19]. More recently, DENV-3 was detected by RT-PCR from a pool of emerging adults collected as larvae during the 2017 outbreak in Côte d’Ivoire [46]. Dengue virus was detected from one pool of F_1_ progeny of a population of *Ae. aegypti* from Nigeria (Lagos), infected with the New Guinea c DENV-2 strain, indicating vertical transmission [63].

## 4 Vector competence studies

The vector competence of field populations of mosquitoes associated with DENV in Africa is poorly characterized (Table 2) in few countries (Figure 2). Except for DENV-2 [64,65,66], few studies have been performed with DENV-1, 3 and 4 [67,68,69]. Vector competence studies were done mainly with sylvatic and domestic *Ae. aegypti* populations from Senegal, Cabo Verde, Kenya, Gabon, Cameroon, Ghana, Nigeria, Republic of Congo and Burkina Faso.

**Table 2:** Mosquito species originated from Africa competent for dengue virus.

Species	Mosquito tested		Titer of the blood meals	Virus serotype (strains; Origin)	Origin	Inf (%)	Diss (%)	Trans (%)	DPE (days)	References
	Country	localities								
*Aedes (Stegomyia) aegypti*	Senegal	Kedougou, Koung Koung, Ndougoubene, Ngoye, Dakar and Barkdji	10^6.5^ and 1.6 × 10^7^ TCID_50_/mL	DENV-2 (ArD 140875; Kedougou)	Sylvatic	0–26	0–75		14	[64]
			10^6.5^ TCID_50_/mL	DENV-2 (ArA 6894; Bobo-Dioulasso)	Epidemic	0–1.85	50–100		14	
		Koung Koung, Kedougou	6.2–8.8 log_10_ TCID_50_/mL	DENV-2 (NGC; New Guinea and 1349; Burkina Faso)	Epidemic	0–25	67–100		14	[70]
			6.2–8.8 log_10_ TCID_50_/mL	DENV-2 (PM33974; Guinea and DakAr2022; Burkina Faso)	Sylvatic	0–3	0–100		14	
		Dakar, St-Louis, Kedougou	4.9 × 10^6^–4.7 × 10^7^ PFU/mL	DENV-1 (SH 29177; Bandia)	Epidemic	71.4–92.5	50–93.8	2.9–5.9	7,15	[67]
			3.5 × 10^6^–2.4 × 10^7^ PFU/mL	DENV-3 (S-162 TvP-3622; Somalia)	Epidemic	62.5–100	50–88.2	3.3–7.5		
			1.2 × 10^6^–2.6 × 10^7^ PFU/mL	DENV-4 (SH 38549; Dakar)	Epidemic	71.4–93.8	56.7–93.8	2.9–20		
		Saint-Louis, Digale, Dakar, Ngoye, Tambacounda, Goudiry, Niemenike, Ngari, PK10, Deux rivieres, Fongolimbi	10^7.5^–8.5 pfu/mL	DENV-2 (JAM1409; Jamaica)	Epidemic		up to 90		14	[65]
		Kedougou, Fatick, Bignona, Richard-Toll, Goudiry, PK10, Mont Rolland, Rufisque	6.02 log_10_ PFU/mL	DENV-2 (75505; Kedougou)	Sylvatic	50–91	29–93		14	[71]
		Dakar, Kedougou	5.10^4.2^ and 5.10^4.4^ MID_50_ ⁄ mL	DENV3 (H87; Hawaii)	Epidemic	2.4–15.2	0–8.3	xx	7, 15,20	[72]
		Dakar, Kedougou	5.10^3.3^ and 5.10^4.3^ MID_50_ ⁄ mL	DENV1_IbH28328; Ibadan)		up to 50	up to 50	xx		
	South Africa	Palm Beach, Durban, Richard Bay, Ndumu, Skukuza	6.1–8.4 Log_10_ MID_50_/mL	DENV-1 (Cassim, Durban)	Epidemic	NA	6–68	50–100	13–20	[80]
			7.0–7.9 Log_10_ MID_50_/mL	DENV-2 (BC 5007, Taipei)	Epidemic	NA	11–64	33–100	14–20	
	Cape Verde	Santiago (seven counties)	2 × 10^5^–5 × 10^4^ FFU/mL	DENV-1 (42735; BR PE)	Epidemic	0–27	0–100	0	7,14,21	[68]
			1.4 × 10^5^–2 × 10^5^ FFU/mL	DENV-2 (3808; BR-PE)	Epidemic	50–80	20–93.3	5.0–65.0	7, 14,21	
			1.0 × 10^6^–2 × 10^6^ FFU/mL	DENV-3 (85469; BR-PE)	Epidemic	10–80	50–87.5	0.0–75.0	7,14,21	
			1.0 × 10^6^–2 × 10^6^ FFU/mL	DENV-4 (1385; Brazil)	Epidemic	0–9	0	0	7,14,21	
			10^7^ FFU/mL	DENV-2 (D2S32; Bangkok)	Epidemic		41.6	8.3	14	[73]
			10^7^ FFU/mL	DENV-3 (Praia; Cabo Verde)	Epidemic		27.3–80	0–20	7,10,14	
	Madagascar	Mahaleja, Joffreville	10^8.2^ MID_50_/mL	DENV-2 (D2S32; Bangkok)	Epidemic	25.0–40.0	xx	x	14	[79]
	Cameroon	19 localities including Douala, Maroua and Yaoundé	10^8.1^ MID_50_ ⁄ mL	DENV-2 (D2S32; Bangkok)	Epidemic		17.2–59.7			[74]
		Yaoundé, Douala, Tibati and Bénoué National Park	10^7^ FFU/mL	DENV-2 (D2S32; Bangkok)	Epidemic	70.8–100	58.8–100	0–50	14,21	[75]
	Kenya	Kilifi, Nairobi	10^5.08^ PFU/ml	DENV-2 (008/01/2012; Mandera)	Epidemic	6.8–21	7.02–42.9		7,14,21	[66]
	Gabon	Franceville	10^8.2^ MID_50_/mL	DENV-2 (D2S32; Bangkok)	Epidemic		52.0, 69.6		14	[76]
	Nigeria	Lagos	0 .001 PFU/mL	DENV-2 (NGC; New Guinea)	Epidemic			37–56		[63]
	Burkina Faso	Kari	0 .001 PFU/mL	DENV-2 (NGC; New Guinea)	Epidemic			0		[63]
	Ghana	Hohoe, Accra, Larabanga, Jirapa	1×10^6^ FFU/mL	DENV-1 (/NIID100/2014; Saitama)		15.4–75.9	0–90.9	0	7,14	[69]
			1×10^6^ FFU/mL	DENV-2 (F299/2017; Ghana)	0–30.2	0–12.5				
	Republic of Congo	Brazzaville	10^7^ ffu/mL	DENV-2 (D2S32; Bangkok)	Epidemic	77.3–95.8	88.2–95.6	36.4	14,21	[75]
*Aedes (Stegomyia) albopictus*	Madagascar,	Anamakia, Antsiranana, Joffreville	10^8.2^ MID_50_/mL	DENV-2 (D2S32; Bangkok)	Epidemic	33.3–93.0	xx	xx	14	[79]
	Gabon	Libreville	10^8^ MID_50_ ⁄ mL	DENV-2 (D2S32; Bangkok)	Epidemic		13, 21.4		14	[77]
	Cameroon	12 localities including Yaoundé and Douala	10^8.1^ MID_50_/mL	DENV-2 (D2S32; Bangkok)	Epidemic		13.3–47.5		14	[74]
		Yaoundé, Douala, Tibati							14,21	[75]
	Republic of Congo	Brazzaville	10^7^ ffu/mL	DENV-2 (D2S32; Bangkok)	Epidemic				14,21	[75]
	La Reunion Island	10 localities including La Marine, Sainte Marie, Saint Pierre	10^8.2^ MID_50_/mL	DENV-2 (D2S32; Bangkok)	Epidemic	18–52			14	[78]
*Ae. (Diceromyia) furcifer*	Senegal	Kedougou	6.5–9.2 TCID_50_/mL	DENV-2 (NGC; New Guinea and 1349; Burkina Faso)	Epidemic	58–94	22–75		14	[70]
		Kedougou	7.0–8.0 TCID_50_/mL	DENV-2 (PM33974; Guinea and DakAr2022; Burkina Faso)	Sylvatic	26–97	0–48			
		Kedougou	1.6 × 10^6^	DENV-4 (Haiti73; Haiti)	Epidemic	88.5	84.6	0		[67]
			3.1 × 10^6^	DENV-3 (Carec 01–11828; Barbados)	Epidemic	86.6	77.8	0		
	South Africa	Mica	6.8–7.1 Log_10_ MID_50_/mL	DENV-1 (Cassim; Durban)			0–3	0	15,17	[80]
		Mica, Ndumu	8.2–8.4 Log_10_ MID_50_/mL	DENV-2 (BC 5007; Taipei)			9, 17	50	15–18	
*Ae. (Stegomyia) luteocephalus*	Senegal	Kedougou	5.5–8.2 TCID_50_/mL	DENV-2 (NGC; New Guinea and 1349; Burkina Faso)	Epidemic	0–89	50			[70]
			5.5–9.2 TCID_50_/mL	DENV-2 (PM33974; Guinea and DakAr2022; Burkina Faso)	Sylvatic	58–79	13–27			
			1.4 × 10^6^ PFU/mL	DENV-4 (SH 38549; Dakar)	Epidemic	77.3	72.7	4.5	7,15	[67]
*Ae. (Diceromyia) taylori*		Kedougou	4.9 × 10^6^ PFU/mL	DENV-1 (SH 29177; Bandia)	Epidemic	68	64	0		[67]
			3.5 × 10^6^ PFU/mL	DENV-3 (S-162 TvP-3622; Somalia)	Epidemic	76.7	76.7	6.7	7,15	
			2.6 × 10^7^ PFU/mL	DENV-4 (SH 38549; Dakar)	Epidemic	83.9	77.4	0		
*Ae. (Stegomyia) streliziae*	South Africa	Palm Beach	7.1	DENV-1 (Cassim; Durban)	Epidemic		33	0	12,14	[80]
		Palm Beach, Armadale	7.1–7.8	DENV-2 (BC 5007; Taipei)	Epidemic		54, 60	29	14–16	
*Ae. (Aedimorphus) vittatus*	Senegal	Kedougou	5.8 TCID_50_/mL	DENV-2 (NGC; New Guinea)	Epidemic	0			14	[70]
			6.5–8.8 TCID_50_/mL	DENV-2 (PM33974; Guinea and DakAr2022; Burkina Faso)	Sylvatic	6 and 19	60, 100			

TCID_50_/mL: 50% tissue culture infectious doses; MID_50_/mL: 50% of mosquito infectious dose for *Ae. aegypti*; PFU/ml: Plaque formed unit; ffu/ml: Foci-Formed unit; Inf: infection rate; Diss: Dissemination rate; Trans: transmission rate; DPE: days post exposure when mosquitoes were tested.

Populations of *Ae. aegypti* from Nigeria (Lagos) and Burkina Faso (Kari) infected with a DENV-2 (New Guinea c strain) by intrathoracic inoculation were able to transmit the virus to suckling mice [63]. The first study by Diallo et al. [70] showed a low susceptibility of two populations of *Ae. aegypti* from Senegal to an epidemic and sylvatic DENV-2 strains. In this study, the infection and dissemination rates ranged between 0-25% and 67–100%, respectively. The low susceptibility of six populations of *Ae. aegypti* from Senegal, from different bioclimatic zones, to DENV-2 (a sylvatic and an epidemic strain) was confirmed later by Diallo et al. [64]. After 14 days post exposure, they found that these populations of *Ae. aegypti* had low infection rates (0–26%) and dissemination rates ranging between 10 and 100%. The authors did not find geographic variations of the vector competence of *Ae. aegypti* for DENV-2 in this study. The low infection rates obtained during these studies have been explained, among others, by the virus titer used and/or the genetic variability of populations used [64]. Later, 11 populations of Senegalese *Ae. aegypti* were challenged with a DENV-2 strain from Jamaica, and showed a northwest-southeast cline of susceptibility [65]. The main finding of these authors was that the northwestern *Ae. aegypti aegypti* populations had higher disseminated infection rates than the southeastern *Ae. aegypti formosus* populations. These results were discordant with those of a following study using a DENV-2 strain isolated from *Ae. luteocephalus* from Senegal to test 8 populations of *Ae. aegypti* collected across Senegal. Indeed, all these populations, including those from the southeastern part of the country, showed high infection and dissemination rates [71] highlighting the role that the viral isolate plays in vector competence. After the first DENV-3 outbreak that occurred in Dakar, Senegal, in 2009, the ability of *Ae. aegypti* populations from Dakar and Kedougou to transmit DENV-1 and 3 was evaluated [72]. The results showed low susceptibility to DENV-3 but high infection and dissemination rates with DENV-1. However, the oral DENV doses used were low and transmission potential was not tested. This study was followed by a larger vector competence study using several mosquito species including one sylvatic and 2 urbans *Ae. aegypti* populations from Senegal for DENV-1, DENV-3 and DENV-4 using experimental oral infection [67]. The virus was detected in the saliva of all three populations. These results indicated that these *Ae. aegypti* populations are more susceptible to DENV-3 than the other serotypes.

A study of *Ae. aegypti* populations from Nairobi and Kilifi, in Kenya, showed that 12.6 % of the 1117 mosquitoes tested with DENV-2 were infected. The population from Nairobi had a significantly higher infection rate with 16.8% of the mosquitoes with a detectable midgut infection. Mosquito infection rates were higher in high temperatures for both populations, indicating an impact of temperature in the *Ae. aegypti* susceptibility to DENV-2 [66]. In Cabo Verde, the susceptibility of *Ae. aegypti* population from Santiago Island showed moderate susceptibility for DENV-3 and low susceptibility for DENV-2 [73]. This Santiago population was later challenged with the four serotypes of DENV and tested for infection, dissemination and transmission after 7, 14, and 21 days post-infection. They showed high vector competence for DENV-2 and 3 and a low susceptibility to the other serotypes [68]. The vector competence of four populations of *Ae. aegypti* (one urban, one semi-urban and two rural) from Ghana were tested for DENV-1 and 2 isolated from human in Japan for DENV-1 and Ghana for DENV-2 [69]. The urban and semi-urban populations were more susceptible to DENV-1 (45 and 41%, respectively) compared to DENV-2 (4 and 3%, respectively) while the rural populations were refractory to both DENV serotypes tested. The highest dissemination rate (92% at 14 dpi) was observed in the urban population for DENV-1. The other dissemination rates for DENV-1 were 29% in the semi-urban, 27 and 0% in the rural populations. None of the populations disseminated the DENV-2 strain. Around 35% of the urban population and 20% of the semi-urban population that disseminated the DENV-1 had infectious saliva at 14 dpi.

The oral susceptibility of 19 populations of *Ae. aegypti* from Cameroon to a DENV-2 virus strain isolated from a human sample in Bangkok, Thailand, showed statistically different disseminated infection rates ranging from 17.2 to 59.7% [74]. Later, 3 populations of *Ae. aegypti* from rural and urban localities of Cameroon and one population of the Republic of Congo (Brazzaville) orally infected with the same DENV-2 strain, showed a competence to transmit the virus [75]. A population of *Ae. aegypti* from Franceville, Gabon, orally tested using the same DENV-2 strain, showed after 14 days post exposure, infection rates of 52 and 69.6% for the F_2_ and F_3_ generations, respectively [76].

Several populations of *Ae. albopictus* from Gabon, La Reunion Island, Madagascar, Republic of Congo, and Cameroon were infected with a DENV-2 virus strain isolated from a human sample in Bangkok, Thailand. Two populations from the city Libreville, Gabon, showed low dissemination rates of 13 and 21.4% [77]. Ten populations from La Reunion Island showed variables infection rates ranging between 18 and 59% [78]. These infection rates varied geographically and were higher in the east coast of the island, while the disseminated infection rates of the 12 populations from Cameroon that ranged between 13.3 and 47.5% and were comparable [74]. Populations of *Ae. albopictus* collected from Cameroon (3 populations) and the Republic of Congo (1 population from Brazzaville) in 2017–2018, orally infected with a DENV-2 strain from Bangkok, were all able to transmit the virus [75]. Three populations from Madagascar showed susceptibility to the virus ranging between 33.3 and 93% [79].

The first vector competence study on sylvatic DENV vectors from Senegal showed high susceptibility of *Ae. furcifer* (infection rates: 26–97%; dissemination rates: 0–75%) and *Ae. luteocephalus* (infection rates: 0–89%; dissemination rates: 13–50%) to infection by the sylvatic and epidemic DENV-2 strains [70]. *Ae. vittatus* also showed low infection (0–19%) but high dissemination rates (60–100%) to DENV-2 during this study. Following the first DENV-3 outbreak in Senegal in 2009, another study tested the vector competence of *Ae. furcifer* (for DENV-3 and 4), *Ae. taylori* (for DENV-1, 3 and 4) and *Ae. luteocephalus* (for DENV-4) populations from Senegal [67]. The infection and dissemination rates of *Ae. furcifer* varied, between 86.6 to 88.5% and 77.8–84.6%, respectively. Like *Ae. furcifer*, the infection and dissemination rates of the other sylvatic vectors were high for all serotypes tested. Only *Ae. luteocephalus* (for DENV-4; 4.5%) and *Ae. taylori* (for ENV-3; 6.7%) transmitted the virus among sylvatic species tested. Populations *Ae. furcifer* and *Ae. streliziae* from South Africa were susceptible to DENV-1 and 2 with head squash infection rates varying between 0 and 17% for *Ae. furcifer* and 33–60% for *Ae. streliziae* [80]. The transmission rates were 0–50% for *Ae. furcifer* and 0–29% for *Ae. streliziae*.

Thus, vector competence data indicate great variability in the susceptibility of mosquito vectors to DENVs. This variability has been observed between populations of different geographic origins, and for the same population with different viral strains. The influence of the temperature on *Ae. aegypti* vector competence to DENVs was also showed [66].

## 5 Vector control

Very little data is available on vector control efforts during outbreaks in Africa. Vector control during dengue outbreaks in Africa relay on several interventions including insecticides spraying, sources reduction and larviciding [81]. Insecticide spraying in and around houses of dengue positive cases has been widely used during dengue outbreaks in Senegal, Cabo Verde and Côte d’Ivoire. Massive space spraying of insecticides outdoors in the neighborhood or entire city of positive cases has been the most widely used control method used in Senegal, Mauritania, Cabo Verde and Côte d’Ivoire. The susceptibility status of the targeted vector populations to the used insecticides were generally not studied [81]. Insecticides susceptibly tests should be always done before insecticide spraying operations [82]. To the best of our knowledge, larviciding using abate and larvivorous fishes to control dengue vectors were only used in Cabo Verde. Domiciliary visits with social sensitization associated with the removal of breeding sites were implemented during the 2018 dengue outbreak in Fatick and Touba, Senegal. The effectiveness of these interventions has never been evaluated.

## 6 Knowledge gaps and prospects for the future

Nationwide distribution, vector competence and insecticides susceptibilities status of *Ae. aegypti* and other dengue vectors remain largely unknown for most African countries. These questions deserve urgent attention of the medical entomology community while urban dengue epidemics number and frequency are increasing in Africa.

It is also essential to study the bio-ecology of mosquito populations, in particular *Aedes* populations in urban areas in Africa. Most of the data currently available has been obtained in the framework of epidemic investigations [27, 46]. However, these investigations are punctual and usually take place at times when the dynamics and diversity of the vectors do not necessarily reflect the situation that caused the epidemic. A simplified dichotomous morphological key of dengue vectors is needed for the rapid identification of these species in the field. This key will allow scientists involved in dengue studies and mosquito control personal to more rapidly characterize mosquito populations, assess the epidemic risk, and respond efficiently to dengue outbreaks.

It would also be very useful to investigate the spatial distribution of vectors and the environmental and socio-economic risk factors associated with epidemic dynamics in order to propose targeted, efficient and sustainable control strategies [83]. The control strategies usually implemented during epidemics have never been monitored and evaluated. New innovative control strategies need to be developed and evaluated. These strategies should ideally use a package of low-cost technologies developed in Africa. These technologies must be produced and maintained at local and national levels. Strategies must be easy to understand, culturally acceptable and not labor intensive to implement by local communities. Finally, the efficacy and efficiency of these strategies must be regularly monitored and evaluated by public health authorities.

There is very little data available on other *Aedes* species present in urban areas in Africa that could play an important role in dengue transmission. With the progressive urbanization and gradual destruction of the forests, which are the natural habitats of the sylvatic vectors, it is essential to examine the evolution of these sylvatic *Aedes* to understand how they will adapt to the urban environment. Until now, DENV-2 is the main serotype detected in the forest environment in Africa [53]. The possibility of DENV-1, 3, and 4 to invade the forest environment and establish sylvatic cycles is unknown and should be investigated. Adaptation of sylvatic *Aedes* into a more urban environment and DENV-1,3 and 4 to the sylvatic environment could complicate the epidemiology of dengue fever in Africa and result in more frequent outbreaks. Finally, it is essential to study the vector competence of the *Ae. aegypti*, *Ae. albopictus* and all *Aedes* species populations in all African cities where they are abundantly present for all dengue serotypes from Africa, Asia and the Americas.

## 7 Concluding remarks

The objective of this paper was to review available data on dengue vectors in Africa. Evidences incriminated *Ae. aegypti* as a dengue vector in the urban setting in several countries in Africa (Senegal, Nigeria, Burkina Faso, Cabo Verde, Tanzania, and Kenya), while *Ae. albopictus* was only incriminated in Gabon. The sylvatic cycle of DENV, involving mainly arboreal mosquitoes and non-human primates, was described for DENV-2 in Nigeria, Senegal, Côte d’Ivoire and Burkina Faso. Several *Aedes* species (*Ae. furcifer*, *Ae. taylori*, *Ae. luteocephalus*, *Ae. vittatus*, *Ae. africanus*, *Ae. opok*, *Ae. cumminsii*, *Ae. dalzieli*, and *Ae. aegypti*) were associated with DENV in the sylvatic environment. Detection of the virus from male mosquitoes and adults collected as immatures during field studies in some countries suggested that DENV is probably maintained in nature by vertical transmission.

Vector competence studies done with sylvatic and domestic *Ae. aegypti* populations (from Senegal, Cabo Verde, Kenya, Gabon and Cameroon) showed great variability in the susceptibility of these populations. This variability was geographic origin, viral serotypes, and temperature dependent. Several populations of *Ae. albopictus* from Gabon, La Reunion Island, and Cameroon infected with a DENV-2 virus showed variables susceptibilities. Vector competence studies on sylvatic DENV vectors from Senegal, showed high susceptibility of *Ae. furcifer* and *Ae. luteocephalus* to infection. *Ae. vittatus* also showed low infection but high dissemination rates to DENV-2. The infection and dissemination rates of *Ae. taylori* were also high for all serotypes tested.

Data on distribution, vector competence and insecticide susceptibilities status of *Ae. aegypti* and other dengue vectors are lacking for most African countries. These questions need to be investigated. Specifically, studies on the bio-ecology of *Aedes* species in urban areas are needed. A simplified dichotomous key of dengue vectors will be helpful for that purpose. The other questions that need to be investigated include environmental and socio-economic risk factors associated with dengue vectors dynamics, control strategies and adaptation of vectors and viral serotypes to new environments.

## Declarations

### Author contribution statement

Diawo Diallo, Babacar Diouf, Alioune Gaye, El hadji NDiaye, Ndeye Marie Sene, Ibrahima Dia and Mawlouth Diallo: Conceived and designed the experiments; Performed the experiments; Analyzed and interpreted the data; Contributed reagents, materials, analysis tools or data; Wrote the paper.

### Funding statement

This research did not receive any specific grant from funding agencies in the public, commercial, or not-for-profit sectors.

### Data availability statement

Data included in article/supplementary material/referenced in article.

### Declaration of interests statement

The authors declare no conflict of interest.

### Additional information

No additional information is available for this paper.
